# Rare manifestation of a large stenosing gastrointestinal tumor caused by *Mycobacterium tuberculosis* in a previously healthy man from Austria

**DOI:** 10.1007/s10354-021-00887-x

**Published:** 2021-10-06

**Authors:** Guangyu Shao, Bakari Chitechi, Gamze Demireli, Karoline Ornig, Matthias J. Neuböck, Sven Heldt, Michael Mandl, Christian Paar, Markus Winkler, Bernd Lamprecht, Helmut J. F. Salzer

**Affiliations:** 1 Department of Pulmonary Medicine, Kepler University Hospital, Krankenhausstr. 9, 4021 Linz, Austria; 2 Department of General Surgery, Kepler University Hospital, Linz, Austria; 3 Institute of Pathology, Kepler University Hospital, Linz, Austria; 4 Institute of Laboratory Medicine, Kepler University Hospital, Linz, Austria; 5 Faculty of Medicine, Johannes-Kepler-University, Linz, Austria

**Keywords:** Extrapulmonary tuberculosis, TB, Cecum, Telephone consultation, Europe

## Abstract

**Background:**

Gastrointestinal tuberculosis (TB) is a rare manifestation in low TB-incidence countries such as Austria. It is usually seen in immunocompromised patients or in migrants being more susceptible for extrapulmonary disease manifestations.

**Case description:**

We report a very rare manifestation of severe gastrointestinal TB in a 49-year-old previously healthy man from Upper Austria. Endoscopy showed a large tumor mass obstructing about 2/3 of the lumen of the cecum. Positron emission tomography/computed tomography scan revealed not only a high metabolic activity in the tumor mass, but also active pulmonary lesions in both upper lung lobes. Bronchial secretion showed acid-fast bacilli in the microscopy and polymerase chain reaction was positive for *M. tuberculosis* complex. Phenotypic resistance testing showed no resistance for first-line anti-TB drugs. Treatment with isoniazid, rifampicin, pyrazinamide and ethambutol was initiated. Based on therapeutic drug monitoring, the standard treatment regime was adapted to rifampicin high dose. TB treatment was well tolerated and the patient achieved relapse-free cure one year after the end of treatment.

**Conclusion:**

Gastrointestinal involvement mimicking an intestinal tumor is a very rare TB manifestation in previously healthy Austrians. However, it should be kept in mind due to increasing migration from countries with higher rates of extrapulmonary TB and due to an increasing number of immunocompromised patients. TB telephone consultations can support medical professionals in the diagnosis and the management of complex TB patients. TB management is currently at a transitional stage from a programmatic to personalized management concept including therapeutic drug monitoring or biomarker-guided treatment duration to achieve relapse-free cure.

## Introduction

Tuberculosis (TB) accounts for one of the major global health problems accounting for around 10 million newly infected individuals with about 1.4 million deaths in 2019 [1]. *Mycobacterium tuberculosis* can cause pulmonary as well as extrapulmonary disease. The latter may affect any organ accounting for around 16% of all TB cases worldwide [1].

The prevalence of extrapulmonary TB varies significantly by geographic region and ethnicity [2]. In countries with a low TB-incidence such as Austria extrapulmonary TB is usually seen in immunosuppressed patients or in foreign-born individuals from Middle Eastern, Asian or African countries [2–5]. Other risk factors for abdominal TB are human immunodeficiency virus (HIV) infection, malnutrition, diabetes mellitus, malignancy, liver cirrhosis, peritoneal dialysis, and certain biologics including tumor necrosis factor-alpha (TNF-α) inhibitors [6, 7]. Abdominal TB can involve the gastrointestinal tract, peritoneum, lymph nodes, or any solid organ accounting for approximately 12% of extrapulmonary TB [5]. It may present as ulceration, peritoneal seeding, tumor mass, or abscess formation mimicking other abdominal diseases [8–10]. Establishing the diagnosis is often challenging, as clinical, radiological and laboratory findings are nonspecific and the performance of diagnostic tests is limited [10, 11].

According to the most recent TB report of the Austrian Agency for Health and Food Safety (AGES), nearly 25% of TB cases in 2018 were extrapulmonary manifestations. However, information about ethnicity and site of disease was not specified [12]. However, in low TB-incidence countries abdominal TB of the gastrointestinal tract appears to be extremely uncommon in previously healthy Caucasian individuals [5, 10].

We report a very rare manifestation of severe abdominal TB mimicking a large intestinal tumor in a previously healthy Caucasian man born in Upper Austria.

## Case description

A 49-year-old previously healthy man from Upper Austria presented to the general practitioner with occasional epigastric pain aggravating after eating a meal. In physical examination a painless and soft abdominal mass was palpable in the right lower abdomen. Routine laboratory findings were unremarkable. An abdominal ultrasound showed a stenosis of the ascending colon.

**Fig. 1:**
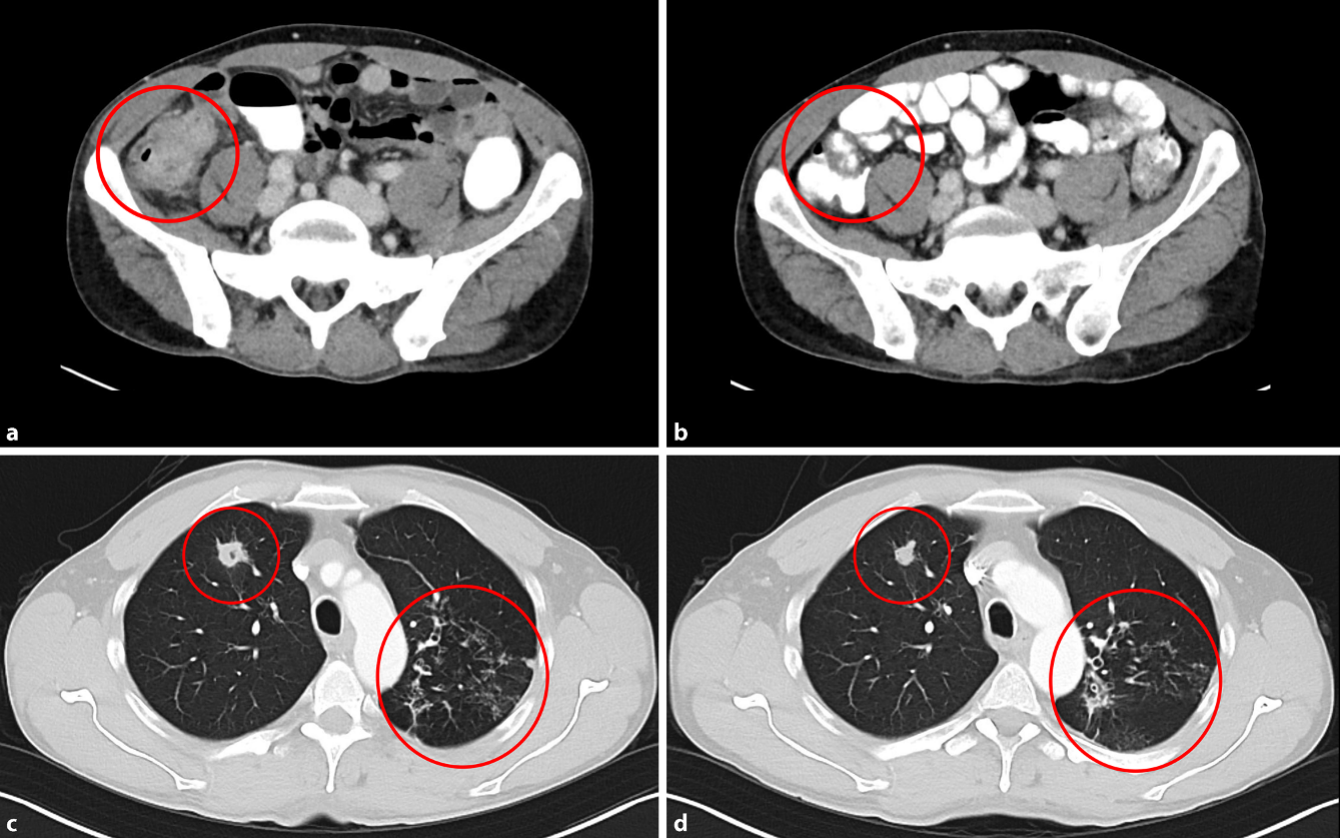
CT scan of the abdomen and the chest showing a gastrointestinal tumor mass in the cecum (*red circle*) and a pulmonary nodule in the left upper lung lobe as well as infiltrates in the right upper lung lobe (*red circles*) at time of diagnosis (**a,** **c**) and after 3 months of antimycobacterial treatment, respectively (**b,** **d**)

**Fig. 2:**
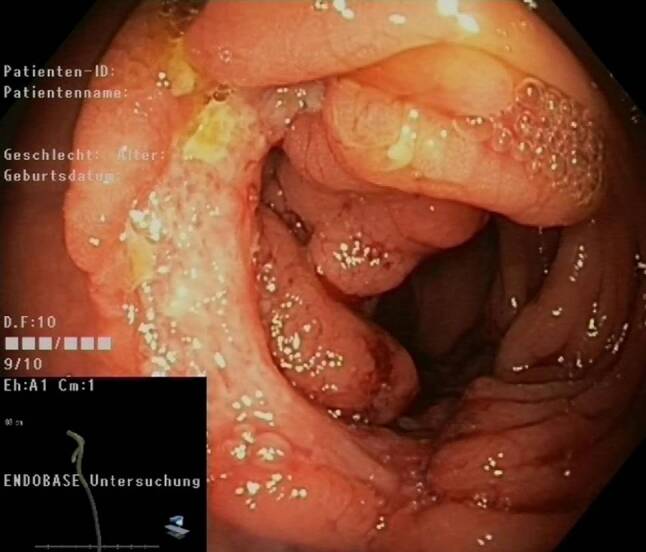
Endoscopic image showing a gastrointestinal tumor mass with about 10 cm in length obstructing about 2/3 of the lumen of the cecum

**Fig. 3:**
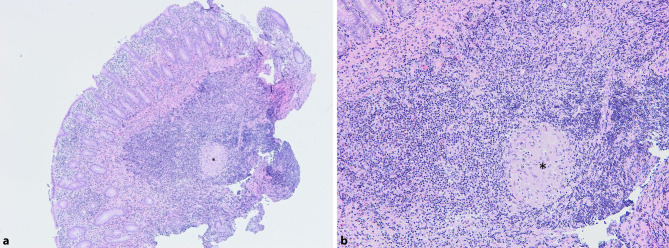
Histopathological specimen obtained from the tumor mass in the cecum showing intestinal mucosa with a highly active and chronic inflammation with epithelioid granuloma (*star*) and giant cells (hematoxylin and eosin; magnification: **a **×4 and **b** ×20)

Computed tomography (CT) scan of the abdomen revealed marked thickening of the ascending colon wall with stenosis of the lumen and a retroperitoneal lymphadenopathy (Fig. 1a). For further investigation a colonoscopy was performed showing a tumor mass obstructing about 2/3 of the lumen of the cecum (Fig. 2). A biopsy was taken for histopathological evaluation. Gastrointestinal malignancy was suspected and the patient was subsequently registered for discussion in the interdisciplinary tumor board. Meanwhile, results of the histopathological evaluation revealed epithelioid granuloma with giant cells and surrounding inflammatory cells without any evidence of malignancy (Fig. 3). This finding was highly suspicious for *M. tuberculosis* infection.

The patient was transferred to the infectious disease ward of the Kepler University Hospital in Linz, Austria. He was isolated in a single-occupancy patient-care room and sputum samples were collected. CT scan of the chest showed a nodule with a small cavity in the upper lobe of the right lung and infiltrates on the left side (Fig. 1c). Repeated smears for acid-fast bacilli were negative, but polymerase chain reaction (PCR) assay testing for *M. tuberculosis* complex (GeneXpert MTB/RIF assay, Cepheid, Sunnyvale, CA, USA) from two different sputum samples were positive. TB diagnosis was reported to the TB control office of the Magistrat Linz were we received the information that his father had died 6 years before due to miliary TB. Further TB contact tracing identified another TB infected person from his close contacts.

Molecular susceptibility testing for rifampicin and isoniazid resistance was not possible due to the low mycobacterial load in the sputum. Consequently, a bronchoscopy was performed. Analyses of bronchial secretion showed acid-fast bacilli in the microscopy and PCR was again positive for *M. tuberculosis* complex. Time to culture positivity was 9 days showing no phenotypic resistance against rifampicin, isoniazid, ethambutol, pyrazinamide, and streptomycin. Viral serologic testing was negative for HIV-1/2.

**Fig. 4:**
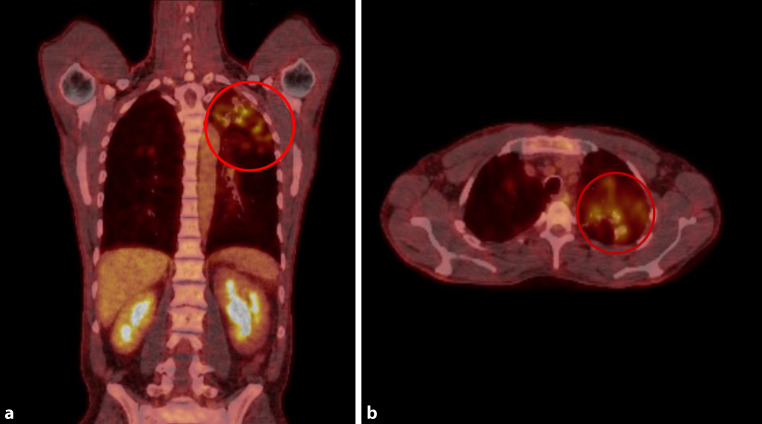
PET CT scan images of coronal (**a**) and axial (**b**) planes showing high metabolic activity in the pulmonary lesions in the left upper lung lobe (*red circles*) with a maximum standardized uptake value (SUVmax) of 4.0–6.4. The high metabolic activity in the abdomen is a normal finding and represents the kidneys

**Fig. 5:**
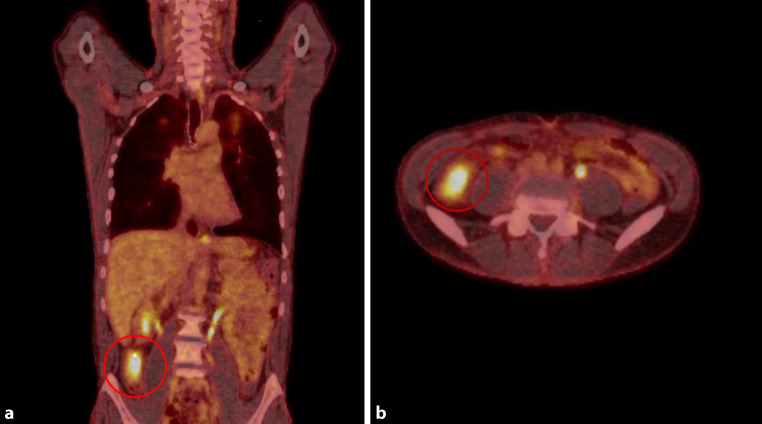
PET CT scan images of the coronal (**a**) and axial (**b**) planes showing high metabolic activity in the tumor mass of the cecum (*red circles*) with a maximum standardized uptake value (SUVmax) of 7.9. The high metabolic activity cranial of the tumor in coronal view and at the opposite side of the axial plane is a normal finding and represents parts of the kidney or urinary tract

Antimycobacterial therapy was initiated including isoniazid (H) 300 mg once daily (od), rifampicin (R) 600 mg od, pyrazinamide (Z) 1500 mg od (25 mg/kg body weight) and ethambutol (E) 1000 mg od (15 mg/kg body weight). To rule out involvement of other organs a whole body positron emission tomography/computed tomography (PET-CT) scan with F‑18 fluorodeoxyglucose ([18F]FDG; Siemens Biograph 40, Siemens Healthcare AG, Lausanne, Switzerland) was performed showing increased metabolic activity in the apical regions of both upper lung lobes as well as in the cecum compatible with the tumor mass (Fig. 4 and 5). Other organs did not show any pathological tracer uptake. Contrast magnetic resonance imaging scan (Magnetom Skyra, Siemens Healthcare AG, Lausanne, Switzerland) of the brain showed no evidence of cerebral involvement.

Treatment was well tolerated without any adverse events. Therapeutic drug monitoring revealed a suboptimal maximal concentration (C_max_) of rifampicin with 4.4 mg/l two hours post-dose. Consequently rifampicin dose was increased to 1200 mg od resulting in a sufficient C_max_ of 16.0 mg/l [13, 14]. On the first follow-up CT scan of the abdomen and chest 3 months after the start of antimycobacterial therapy, improvement was documented showing a significantly shrinking intestinal tumor mass compared to initial findings (Fig. 1). Treatment with isoniazid and high-dose rifampicin was continued for another 7 months before TB treatment was stopped after a total of 12 months. At the end of treatment, the colonoscopy showed no pathological findings.

## Discussion

We report a rare case of pulmonary and abdominal TB presenting with a large stenosing gastrointestinal tumor in a previously healthy man from Upper Austria. Gastrointestinal TB is usually seen in immunocompromised patients or in migrants coming from Middle East, Asia or Africa [5, 10, 15]. However, our patient was neither immunocompromised nor born abroad. Based on the total number of TB patients in Austria, the proportion of extrapulmonary TB increased considerably from 17 to 25% between 2008 and 2018, while the rate of pulmonary TB patients decreased continuously [12, 16]. This could be explained by an increasing number of migrants being more susceptible for developing extrapulmonary TB. Likewise the percentage of TB patients born abroad increased from 40 to 67% in Austria during the same time period [12, 16], but it has not been reported how many of these patients contributed to the extrapulmonary cases. Therefore, we also did not find any numbers grouped by country of origin for gastrointestinal TB cases in Austria. In other countries such as in Saudi Arabia gastrointestinal TB is the most common type of extrapulmonary infection representing 16% of all TB manifestations [17]. In India and South Africa gastrointestinal TB is the second leading cause of malabsorption [10]. The reasons for these geographical differences are not fully understood and may be partly explained by differences in socioeconomic status, eating habits and nutrition, prevalence of HIV/AIDS and TB [10].

Certain drugs and diseases weakening the immune system are additional risk factors for developing gastrointestinal TB. Especially treatments with TNF‑α inhibitors increase the risk for TB significantly. The relative risk is up to 25 times higher [7]. Extrapulmonary manifestations occur significantly more often among TB patients who received anti-TNF‑α therapy ranging from 56 to 75% [18, 19]. It is therefore crucial to screen individuals for latent TB infection (LTBI) before starting anti-TNF‑α therapy by tuberculin skin test or interferon gamma assays. If screening for LTBI is positive, active TB disease has to be excluded (e.g., chest X‑ray, sputum) and an LTBI treatment has to be initiated before starting anti-TNF‑α therapy [7, 18, 19].

Also HIV/AIDS patients are more likely to develop extrapulmonary TB with a relative risk up to seven times higher compared to HIV-negative individuals [11]. An Indian autopsy study reported that 14% of deceased AIDS patients have gastrointestinal TB [20]. Given the fact that both diseases are closely associated, the World Health Organization (WHO) recommends to actively screen for TB symptoms among HIV patients and to test TB patients for HIV [1].

Moreover, certain human genetic factors have been found to be a risk factor for developing extrapulmonary TB. Single nucleotide polymorphisms identified in a variety of genes have been shown to compromise the immune system, predisposing for mycobacterial infection [21]. An example is the P2X_7_ receptor on macrophages that induces elimination of internalized mycobacteria in case of activation. A loss-of-function polymorphism of the P2X_7_ gene is found in south-east Asians, which is associated with an impaired function of macrophages increasing the susceptibility to extrapulmonary TB [22]. Furthermore, strains of *M. tuberculosis* show genetic differences between geographic regions. Therefore, a pathogen-related susceptibility for extrapulmonary TB may also play a role in addition to different host factors [9, 23].

The diagnosis of abdominal TB is challenging and is often being mistaken for inflammatory bowel disease or malignancy in low TB-incidence countries as demonstrated by our patient [5, 15, 24]. Radiological findings are unable to confirm the diagnosis, but may suggest gastrointestinal TB indicating abdominal lymphadenopathy and/or thickening of the intestinal wall. Abdominal lymphadenopathy is most frequently located in the parapancreatic, mesenteric or paracaval region [10]. Microbiological confirmation requires appropriate specimens. Biopsy specimens are frequently fixed and immersed in formalin or other preserving agents causing destruction of mycobacteria, which often makes the sample inadequate for culture and may hamper PCR analysis [25]. Therefore, it is important to obtain native tissue samples to enable appropriate diagnostic test results, whenever gastrointestinal TB is suspected. Beside routine histopathological evaluation, microbiological diagnostics should always include smear microscopy (e.g., Ziehl–Neelsen staining), solid and/or liquid TB cultures (e.g., Lowenstein–Jensen agar), and nucleic amplification tests (e.g., PCR) for detection of *M. tuberculosis* complex [9–11, 26]. However, histopathological evaluation and mycobacterial cultures from tissue are reported to have a limited diagnostic yield lacking sensitivity and specificity. In a Korean study of 225 patients with gastrointestinal TB, necrosis was only present in 11% and microscopic detection of acid-fast bacilli was only possible in 17% of cases. Growth of mycobacterial culture was reported in 52 of 177 patients corresponding to a sensitivity of only 23% [27]. In contrast *M. tuberculosis* complex PCR from biopsy specimens has been reported to have a sensitivity of over 90% and specificity of 100% [26, 28]. Comparable results were reported for the *M. tuberculosis* complex PCR from fecal samples in patients with gastrointestinal TB with a sensitivity of 89% and the specificity of 100% [29]. Unfortunately, in our patient the PCR for *M. tuberculosis* complex was negative from feces. Most TB experts recommend obtaining native biopsy samples from the gut to perform a *M. tuberculosis* complex PCR, although the performance of a PCR from fecal samples seems to be comparable and less invasive.

Guideline-based treatment for gastrointestinal TB is comparable to pulmonary TB consisting of an intensive phase with rifampin, isoniazid, ethambutol and pyrazinamide for 2 months, followed by a continuation phase with rifampicin and isoniazid for another 4–7 months. The optimal treatment duration is currently unclear [10]. Some experts have concerns that the standard treatment duration of 6 months may not always achieve relapse-free cure, while others argue that longer treatment increases the risk of poor treatment adherence, adverse events and higher costs [30]. A systematic Cochrane review by Julien et al. including three randomized controlled trials with a total number of 328 abdominal TB patients comparing 6‑month versus >6-month TB treatment showed no difference in clinical cure at the end of treatment. Additionally no difference was shown in preventing relapse at the end of the follow-up periods. However, the level of evidence remains limited because the trials were small in size and lacked consistency in study design [30]. Nevertheless, the scientific progress indicates that the treatment of TB patients is presently at a transition stage from programmatic to personalized TB management [31, 32]. Personalized TB management based on current evidence will provide opportunities to substantially improve the outcome of patients with tuberculosis [13].

Contact to an experienced TB center can support colleagues in the management of difficult-to-treat TB patients. Professional telephone consultations are provided for free, for example, by the Research Center Borstel in Germany (TBinfo; https://www.dzif.de/en/working-group/clinical-tuberculosis-centre) or by the Kepler University Hospital in Austria (TBC Beratungstelefon für Ärztinnen und Ärzte; https://www.kepleruniklinikum.at/versorgung/kliniken/lungenheilkunde/schwerpunkte-und-leistungen/).

## References

[1] WHO (2020). Global tuberculosis report 2020.

[2] Sandgren A, Hollo V, van der Werf MJ (2013). Extrapulmonary tuberculosis in the European Union and European Economic Area, 2002 to 2011. Euro Surveill.

[3] Peto HM, Pratt RH, Harrington TA (2009). Epidemiology of extrapulmonary tuberculosis in the United States, 1993–2006. Clin Infect Dis.

[4] Forssbohm M, Zwahlen M, Loddenkemper R (2008). Demographic characteristics of patients with extrapulmonary tuberculosis in Germany. Eur Respir J.

[5] Stelzmueller I, Bellmann-Weiler R, Klaus A (2009). Abdominal tuberculosis: experience with three cases from an Austrian centre. Eur Surg.

[6] Lazarus AA, Thilagar B (2007). Abdominal tuberculosis. Dis Mon.

[7] Solovic I, Sester M, Gomez-Reino JJ (2010). The risk of tuberculosis related to tumour necrosis factor antagonist therapies: a TBNET consensus statement. Eur Respir J.

[8] Golden MP, Vikram HR (2005). Extrapulmonary tuberculosis: an overview. Am Fam Physician.

[9] Donoghue HD, Holton J (2009). Intestinal tuberculosis. Curr Opin Infect Dis.

[10] Choi EH, Coyle WJ (2016). Gastrointestinal tuberculosis. Microbiol Spectr.

[11] Uygur-Bayramicli O, Dabak G, Dabak R (2003). A clinical dilemma: abdominal tuberculosis. World J Gastroenterol.

[12] BMASGK (2019). Nationale Referenzzentrale für Tuberkulose Jahresbericht 2018.

[13] Salzer HJ, Wassilew N, Köhler N (2016). Personalized medicine for chronic respiratory infectious diseases: tuberculosis, nontuberculous mycobacterial pulmonary diseases, and chronic pulmonary aspergillosis. Respiration.

[14] Onorato L, Gentile V, Russo A (2021). Standard versus high dose of rifampicin in the treatment of pulmonary tuberculosis: a systematic review and meta-analysis. Clin Microbiol Infect.

[15] Kentley J, Ooi JL, Potter J (2017). Intestinal tuberculosis: a diagnostic challenge. Trop Med Int Health.

[16] BMASGK (2011). Nationale Referenzzentrale für Tuberkulose Jahresbericht 2010.

[17] Al Karawi MA, Mohamed AE, Yasawy MI (1995). Protean manifestation of gastrointestinal tuberculosis: report on 130 patients. J Clin Gastroenterol.

[18] Keane J, Gershon S, Wise RP (2001). Tuberculosis associated with infliximab, a tumor necrosis factor alpha-neutralizing agent. N Engl J Med.

[19] Wolfe F, Michaud K, Anderson J (2004). Tuberculosis infection in patients with rheumatoid arthritis and the effect of infliximab therapy. Arthritis Rheum.

[20] Lanjewar DN, Anand BS, Genta R (1996). Major differences in the spectrum of gastrointestinal infections associated with AIDS in India versus the west: an autopsy study. Clin Infect Dis.

[21] Naranbhai V (2016). The role of host genetics (and genomics) in tuberculosis. Microbiol Spectr.

[22] Fernando SL, Saunders BM, Sluyter R (2007). A polymorphism in the P2X7 gene increases susceptibility to extrapulmonary tuberculosis. Am J Respir Crit Care Med.

[23] Te Beek LA, van der Werf MJ, Richter C (2006). Extrapulmonary tuberculosis by nationality, The Netherlands, 1993–2001. Emerg Infect Dis.

[24] Papis D, Branchi V, Gomez L (2015). Abdominal tuberculosis mimicking Crohn’s disease’s exacerbation: a clinical, diagnostic and surgical dilemma. A case report. Int J Surg Case Rep.

[25] Solovic I, Jonsson J, Korzeniewska-Koseła M (2013). Challenges in diagnosing extrapulmonary tuberculosis in the European Union, 2011. Euro Surveill.

[26] Lowbridge C, Fadhil SAM, Krishnan GD (2020). How can gastro-intestinal tuberculosis diagnosis be improved? A prospective cohort study. BMC Infect Dis.

[27] Lee YJ, Yang SK, Myung SJ (2004). The usefulness of colonoscopic biopsy in the diagnosis of intestinal tuberculosis and pattern of concomitant extra-intestinal tuberculosis. Korean J Gastroenterol.

[28] Debi U, Ravisankar V, Prasad KK (2014). Abdominal tuberculosis of the gastrointestinal tract: revisited. World J Gastroenterol.

[29] Balamurugan R, Venkataraman S, John KR (2006). PCR amplification of the IS6110 insertion element of Mycobacterium tuberculosis in fecal samples from patients with intestinal tuberculosis. J Clin Microbiol.

[30] Jullien S, Jain S, Ryan H (2016). Six-month therapy for abdominal tuberculosis. Cochrane Database Syst Rev.

[31] Lange C, Aarnoutse R, Chesov D (2020). Perspective for precision medicine for tuberculosis. Front Immunol.

[32] Heyckendorf J, Marwitz S, Reimann M (2021). Prediction of anti-tuberculosis treatment duration based on a 22-gene transcriptomic model. Eur Respir J.

